# Axon degeneration induces glial responses through Draper-TRAF4-JNK signalling

**DOI:** 10.1038/ncomms14355

**Published:** 2017-02-06

**Authors:** Tsai-Yi Lu, Jennifer M. MacDonald, Lukas J. Neukomm, Amy E. Sheehan, Rachel Bradshaw, Mary A. Logan, Marc R. Freeman

**Affiliations:** 1 Department of Neurobiology, University of Massachusetts Medical School, Worcester, Massachusetts 01605, USA

## Abstract

Draper/Ced-1/MEGF-10 is an engulfment receptor that promotes clearance of cellular debris in *C. elegans*, *Drosophila* and mammals. Draper signals through an evolutionarily conserved Src family kinase cascade to drive cytoskeletal rearrangements and target engulfment through Rac1. Glia also alter gene expression patterns in response to axonal injury but pathways mediating these responses are poorly defined. We show Draper is cell autonomously required for glial activation of transcriptional reporters after axonal injury. We identify *TNF receptor associated factor 4* (TRAF4) as a novel Draper binding partner that is required for reporter activation and phagocytosis of axonal debris. TRAF4 and *misshapen* (MSN) act downstream of Draper to activate c-Jun N-terminal kinase (JNK) signalling in glia, resulting in changes in transcriptional reporters that are dependent on *Drosophila* AP-1 (dAP-1) and STAT92E. Our data argue injury signals received by Draper at the membrane are important regulators of downstream transcriptional responses in reactive glia.

## Abstract (web summary)

The engulfment receptor Draper is known to promote glial activation and phagocytosis of debris upon axonal injury. Lu *et al*. identify TNF receptor associated factor 4 (TRAF4) as a binding partner of Draper, and map out the signalling cascade leading to reactive gliosis in *Drosophila*.

Glial cells are exquisitely sensitive to perturbations of nervous system physiology^1^,^2^,^3^,^4^,^5^,^6^,^7^. In response to neural injury they coordinate a sophisticated cascade of reactive events, including engagement of phagocytic programmes to clear cellular debris, and secretion growth factors to recruit additional immune cells or promote neuronal survival and repair^8^. Reactive glial responses are driven in important ways by changes in glial gene expression following trauma. Strong upregulation of expression of *GFAP* and *S100β* are among the earliest detectable changes in mammalian neural tissues after brain injury, and similar upregulation occurs in chronic neurodegenerative disease^9^,^10^. In peripheral nerves early events include the activation of JNK signalling, which is required for efficient Schwann cell de-differentiation and initiation of regenerative responses^11^. Initiating events in glial reactive responses are presumably driven by ‘injury signals' released by damaged cells that signal to transmembrane receptors on glia, but whether and how cellular debris activates glial transcriptional responses remains mechanistically unclear.

*Drosophila* glia rapidly respond at the transcriptional level to axotomy, which argues reactive glial responses are an ancient feature of the metazoan nervous system^12^. Transcriptional reporters for the sole *Drosophila* STAT molecule, STAT92E, are induced in glial cells within hours after local axons are severed^6^. Axotomy also activates the glial JNK signalling cascade and ultimately transcriptional reporters for the *Drosophila* AP-1 complex (dAP-1)^7^. Activation of STAT92E-dependent transcriptional responses has been shown to require the engulfment receptor Draper^6^, suggesting STAT92E lies genetically downstream of Draper, although how axon degeneration is mechanistically linked to STAT92E- or dAP-1-mediated transcriptional changes is not known.

## Results

### Axon degeneration activates dAP-1 reporters in glia

**Figure 1:**
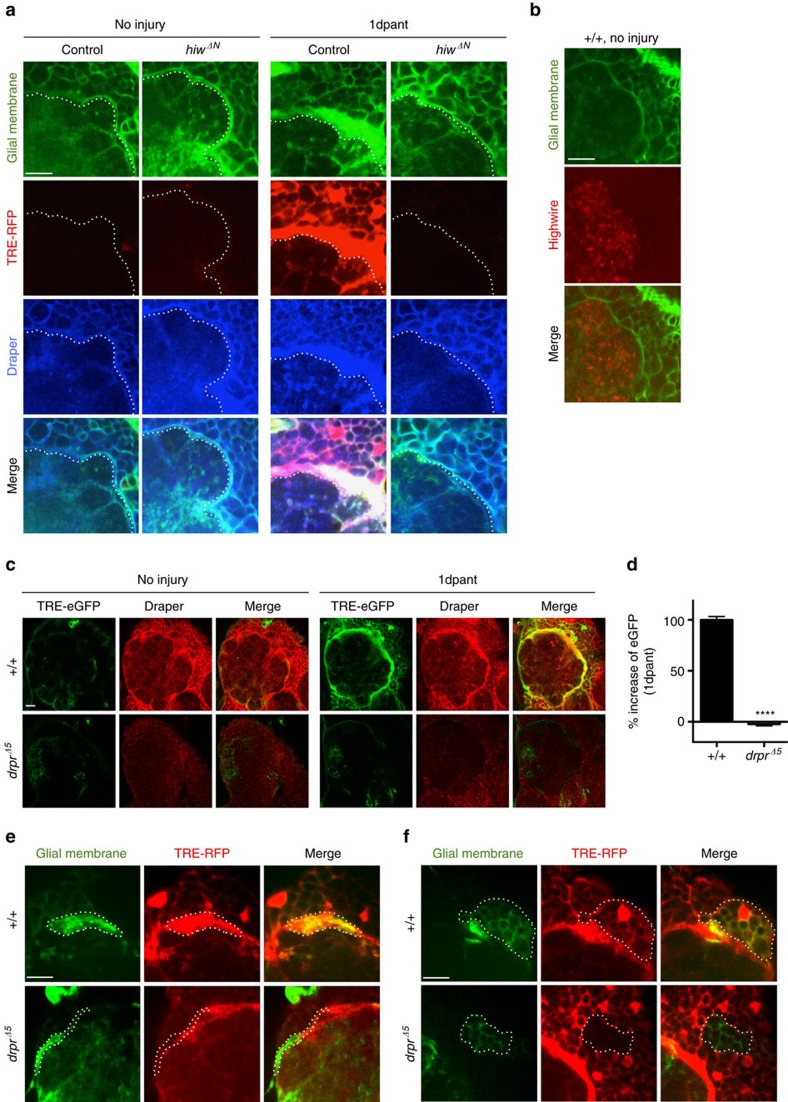
Glial transcriptional activation is downstream of axon degeneration and Draper signalling. (**a**) Glial responses to axon injury in controls and *highwire* null (*hiw*^*ΔN*^) animals after axotomy (1 day after bilateral antennal ablation, 1 dpant). Note glial membrane hypertrophy, activation of the of TRE reporter and upregulation of Draper (anti-Draper) in cohort controls (*hiw*^*ΔN*^*/+; TRE-RFP-16/+; repo-Gal4, UAS-mCD8::GFP/+*). *highwire* null animals (*hiw*^*ΔN*^/Y*; TRE-RFP-16/+; repo-Gal4, UAS-mCD8::GFP/+*), where axon degeneration is blocked, showed no detectable activation of glia. Dotted line delineates edge of the antennal lobe. Representative images were shown from five animals. Scale bar=10 μm. (**b**) Highwire was highly enriched in the synaptic region in the antennal lobe, but not in the surrounding glial cells (labelled with *repo-Gal4, UAS-mCD8::GFP/+*). Representative images were shown from three animals. (**c**) Glia require Draper to activate TRE reporter 1day after antennal ablation. *+/+*: *TRE-eGFP-16; +/+*. *drpr*^*Δ5*^: *TRE-eGFP-16; drpr*^*Δ5*^. Representative images were shown from three animals. Scale bar=10 μm. (**d**) Quantification of the increase of eGFP immunoreactivity in (**c**). *n*=15 each. Unpaired *t*-test, 2-tailed. ****: *P*<0.0001. (**e**) Ensheathing glial MARCM clones (green, outlined) responding to axotomy at day 1 post antennal ablation. Control ensheathing glial clones (+/+) became hypertrophic and exhibited strong TRE reporter activation (red) 1 day after antennal ablation, while *draper* null (*drpr*^*Δ5*^) clones did not respond to axotomy (that is lack of membrane hypertrophy, and activation of TRE reporter) while neighbouring control cells were activated. *+/+*: *TRE-RFP-16, UAS-mCD8::GFP/repo-FLP*^6–2^*; FRT2A, FRT82B/tub-Gal80, FRT2A, repo-Gal4*. *drpr*^*Δ5*^: *TRE-RFP-16, UAS-mCD8::GFP/repo-FLP*^6–2^*; drpr*^*Δ5*^*, FRT2A/tub-Gal80, FRT2A, repo-*Gal4. Representative images were shown from four animals. Scale bar=10 μm. (**f**) TRE-RFP reporter activity in cortex glial MARCM clones (dotted line, green) 1 day after antennal ablation. Similar to ensheathing glia, cortex glial clones that lacked Draper (*drpr*^*Δ5*^) failed to activate TRE reporter. Genotypes as in (**e**). Representative images were shown from four animals. Scale bar=10 μm.

To understand how axonal injury leads to glial transcriptional changes we sought to define the mechanisms regulating activation of the dAP-1 complex in glia after axotomy. We used the dAP-1 transcriptional reporters containing multiple tetradecanoylphorbol acetate response elements (TRE, that is the dAP-1 binding sequence) upstream of either eGFP (*TRE-eGFP*) or RFP (*TRE-RFP*)^13^. Axon injury was induced by surgical removal of adult *Drosophila* third antennal segments, which severs ∼90% of olfactory receptor neurons (ORNs) projecting into the *Drosophila* antennal lobe and axon fragmentation begins within ∼12 h by activation of Wallerian degeneration^2^. Prior to axonal injury *TRE-RFP* was not detectable at significant levels in control animals in any part of the adult brain. However, one day after axonal injury (1 dpant, 1 day post antennal ablation), we observed strong activation of *TRE-RFP* throughout the brain (Supplementary Fig. 1), as previously observed for *TRE-eGFP* (ref. 7), along with glial hypertrophy and increased Draper levels in glia adjacent to the injury site. To determine whether activation of *TRE-RFP* represented a glial response to axon degeneration, we blocked ORN axon degeneration using *highwire* (*hiw*) mutants^14^. Glial cells in *hiw*^*ΔN*^ mutant animals exhibited no morphological changes after axonal injury, *TRE-RFP* activation was not observed in any part of the brain, and Draper levels adjacent to the injury site remained similar to the uninjured controls (Fig. 1a). Hiw is not expressed at detectable levels in glia but is prominent in neurons (Fig. 1b), suggesting the lack of glial activation after axotomy in *hiw* mutants resulted from blockade of axon degeneration, rather than a cell autonomous role for *hiw* in glial activation. Consistent with this notion, pan-neuronal expression of Highwire in *hiw*^*ΔN*^ mutant background rescued glial response to axon degeneration (Supplementary Fig. 2b) while glial expression of a *hiw* cDNA in the *hiw*^*ΔN*^ mutant background did not lead to activation of the *TRE-RFP* reporter (Supplementary Fig. 2a). Activation of the Wallerian degeneration signalling pathway is therefore essential for brain-wide induction of dAP-1 transcriptional reporters in glia after nerve injury. Furthermore, these data indicate glial cells respond specifically to signals generated by degenerating axons, rather than other alterations in axons caused by axotomy (for example loss of neuronal activity).

### Glia activate dAP-1 reporters after injury through Draper

The engulfment receptor Draper activates glial phagocytic function during Wallerian degeneration^2^. We found *draper*^*Δ5*^ null mutant animals also exhibited a striking loss of *TRE-eGFP* induction 1 day after axon injury compared to controls (Fig. 1c,d), indicating that Draper is also required for dAP-1 reporter activation in response to axonal degeneration. ORN axon terminals only project to the antennal lobe. The lack of *TRE-eGFP* activation in *draper* mutants observed throughout the adult brain implied that Draper was required for *TRE-eGFP* activation even in glia at sites distant from degenerating axons in the antennal lobe. To characterize the autonomy of Draper function in activation of dAP-1 transcriptional reporters in brain glia, we generated *draper*^*Δ*5^ mutant clones using the mosaic analysis with a repressible cell marker (MARCM) approach^15^, severed antennal ORN axons, and assayed *TRE-RFP* activation 1 day after axotomy. Based on morphological analysis of clones we determined that activation of *TRE-RFP* occurred in ensheathing (Fig. 1e) and cortex glia (Fig. 1f), but not in astrocytes (Supplementary Fig. 3), consistent with a previous study demonstrating Draper is expressed solely in these glial subtypes in the *Drosophila* adult brain^16^. While control clones in each subtype of glial cell showed strong activation of *TRE-RFP* in response to axonal injury, *draper*^*Δ*5^ mutant clones did not activate the *TRE-RFP* reporter (Fig. 1e,f). This was even true when *draper*^*Δ*5^ mutant clones were made in cortex glia, which are not in direct contact with severed axons (for example Fig. 1f). This was surprising in light of the fact that Draper/Ced-1 is considered a recognition receptor for ‘eat me' cues on the surface of engulfment targets such as axonal debris^1^, which we presumed would require cell–cell contact. This observation suggests that Draper may be activated by a soluble ligand released by severed ORNs, or alternatively that Draper-dependent glial-glial signalling may occur in cortex glia upstream of dAP-1 reporter activation, perhaps similar to MEGF10 signalling in starburst amacrine cells during tiling where MEGF10 appears to act genetically as both a receptor and ligand^17^.

### TRAF4 signals downstream of Draper after axonal injury

**Figure 2:**
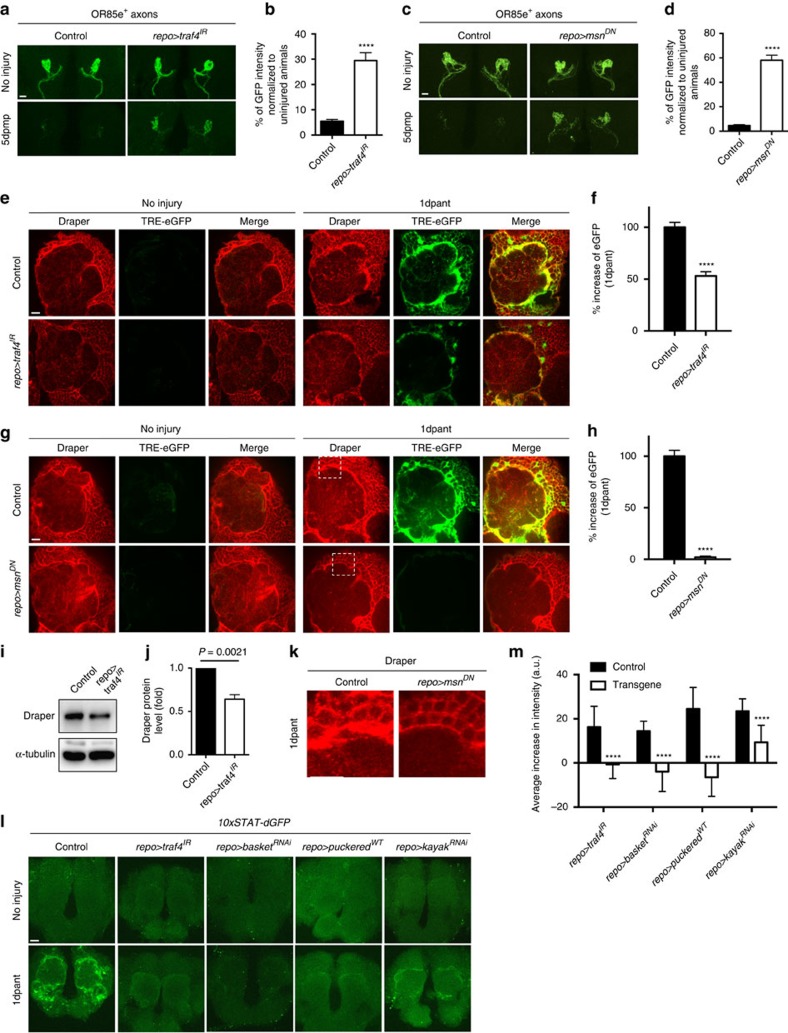
TRAF4 and MSN are upstream of JNK- and STAT92E-mediated responses to axotomy. (**a**) RNAi-mediated depletion of TRAF4 in glia (*repo>traf4*^*IR*^: *OR85e-mCD8::GFP/UAS-traf4*^*IR*^*; repo-Gal4/+*) resulted in a delay in axonal debris clearance 5 days after maxillary malp ablation (5 dpmp) compared to controls (*OR85e-mCD8::GFP/+; repo-Gal4/+*). OR85e^+^ maxillary nerves were labelled with mCD8::GFP. Scale bar for all=10 μm. (**b**) Quantification from (a). *n*=10. Unpaired *t*-test, 2-tailed (for all). (**c**) Expression of dominant-negative MSN (*msn*^*DN*^) suppressed glial clearance of axonal debris. Control: *OR85e-mCD8::GFP, tub-Gal80*^*ts*^*/+; repo-Gal4/+*. *Repo>msn*^*DN*^: *OR85e-mCD8::GFP, tub-Gal80*^*ts*^*/UAS-msn*^*DN*^*; repo-Gal4/+*. Representative images were shown (control: *n*=10; *repo>msn*^*DN*^: *n*=9). (**d**) Quantification from (**c**). (**e**) TRE reporter activity was suppressed in TRAF4 RNAi animals (*repo>traf4*^*IR*^) 1 day after antennal ablation. Control: *TRE-eGFP-16/+; repo-Gal4/+*. *Repo>traf4*^*IR*^: *TRE-eGFP-16/UAS-traf4*^*IR*^*; repo-Gal4/+*. (**f**) Quantification of the eGFP immunoreactivity in (**e**). *n*=25 each. (**g**) TRE reporter activity was inhibited 1 day after antennal ablation by glial expression of MSN^DN^. Control: *TRE-eGFP-16, tub-Gal80*^*ts*^*/+; repo-Gal4/+*. *Repo>msn*^*DN*^: *TRE-eGFP-16, tub-Gal80*^*ts*^*/UAS-msn*^*DN*^*; repo-Gal4/+*. (**h**) Quantification from (**g**). *n*=15 each. (**i**) Draper expression was reduced on western blots when TRAF4 was knocked down in glia from adult dissected central brains. α-tubulin was used as loading control. Representative images were shown from three repeats. Control: *repo-Gal4/+*. *Repo>traf4*^*IR*^: *UAS-traf4*^*IR*^*/+; repo-Gal4/+*. (**j**) Quantification of (**i**). *n*=3. Unpaired *t*-test, 2-tailed. (**k**) Higher magnification images of dashed squares in (**g**) showed the lack of Draper increase 1 day after antennal ablation in *msn*^*DN*^ animals. (**l**) STAT92E-mediated dGFP (degradable GFP) expression was induced in glia 1 day after antennal ablation in control animals (*10xSTAT92E-dGFP/+; repo-Gal4/+*), but blocked in animals where JNK signalling cascade was interrupted by glial expression of *traf4*^*IR*^ (*10xSTAT92E-dGFP/UAS-traf4*^*IR*^*; repo-Gal4/+*), *basket*^*RNAi*^ (*10xSTAT92E-dGFP/UAS-basket*^*RNAi#34138*^*; repo-Gal4/+*) and *puckered*^*WT*^ (*10xSTAT92E-dGFP/+; repo-Gal4/UAS-puckered*), or *kayak*^*RNAi*^ (*repo>kayak*^*RNAi*^:*10xSTAT92E-dGFP/+; repo-Gal4/UAS-kayak*^*RNAi#6212*^). (**m**) Quantification of the increase of dGFP immunoreactivity in (**l**). *Repo>traf4*^*IR*^: *n*=15 each. *Repo>basket*^*RNAi*^, *repo>puckered*^*WT*^ and *repo>kayak*^*RNAi*^: *n*=25 each. Unpaired *t*-test for each group, 2-tailed.

Based on the potent regulation of *TRE-RFP* by Draper, we sought to determine how Draper activation might lead to its activation through dAP-1. We screened a large collection of RNAi lines for novel signalling molecules and assayed for roles in glial clearance of degenerating axons. Interestingly, we found that pan-glial expression of *UAS-traf4*^*IR*^ (ref. 18) resulted in strong suppression of glial clearance of axonal debris, compared to controls (Fig. 2a,b, Supplementary Fig. 4c,d). TRAF4 is known to act upstream in activation of the JNK signalling pathway through association with the MAP kinase kinase kinase kinase (MAP4K) *misshapen* (MSN) (ref. 19), which then phosphorylates the downstream MAP3K Slipper and ultimately drives dAP-1 activation of transcriptional targets^20^. A role for TRAF4 in glial engulfment was intriguing given that we previously implicated a number of downstream components of the JNK signalling pathway in glial engulfment of axonal debris including dAP-1 (ref. 7), but how this pathway might be activated remained unclear.

Pan glial expression of a dominant negative version of MSN (*msn*^*DN*^)^21^ strongly suppressed glial engulfment of axonal debris 5 days after axotomy (Fig. 2c,d). Consistent with a role for TRAF4 and MSN in activation of the JNK signalling pathway we found that glial expression of *traf4*^*IR*^ (Fig. 2e,f) or *msn*^*DN*^ (Fig. 2g,h,k) suppressed activation of the *TRE-eGFP* reporter. Moreover, we observed that depletion of TRAF4 or expression of MSN^DN^ suppressed injury-induced increases in Draper that are normally observed 1 day after axonal injury, and which we previously reported for downstream components of the JNK signalling pathways. We further noted that in *traf4*^*IR*^ animals Draper expression was reduced ∼50% even before axon injury (Fig. 2i,j). This phenotype is similar to glial *stat92e*^*RNAi*^ animals, where Draper levels are reduced in uninjured animals, and engulfment defects can be rescued by overexpression of a *draper* cDNA^6^. In contrast, we found defects in glial responses to axonal degeneration in the *traf4*^*IR*^ background did not result from insufficient Draper levels: re-expression of a functional Draper-I transcript (Supplementary Fig. 4a,b) in *traf4*^*IR*^ animals failed to rescue axonal debris clearance defects (Supplementary Fig. 4c,d). Together these data support a model whereby TRAF4 signals downstream of Draper to promote target engulfment and glial transcriptional reporter activation mediated by STAT92E and dAP-1.

Based on our observation that TRAF4 and MSN regulated injury-induced changes in Draper levels and transcriptional reporters in glia, we sought to define signalling interactions between TRAF4 and MSN, the JNK signalling pathway, and STAT92E. STAT92E activity after axotomy was assayed using the degradable GFP reporter *10xSTAT92E-dGFP* (ref. 22), which is transiently activated in glia 1 day after axotomy^6^. In control uninjured animals, GFP expression was absent in brains, but strongly activated in glia surrounding the antennal lobe 1 day after antennal ORN axotomy (Fig. 2l). Glial expression of *traf4*^*IR*^ suppressed this increase of GFP expression after axon injury (Fig. 2l,m), indicating that TRAF4 is upstream of the injury-induced activation of *10xSTAT92E-dGFP*. Similarly, blockade of the JNK signalling pathway with *basket*^*RNAi*^ (*Drosophila* JNK), or overexpression of the JNK inhibitor Puckered eliminated the injury-induced upregulation of *10xSTAT92E-dGFP* (Fig. 2l,m). Finally, knockdown of Kayak, a component of the dAP-1 transcriptional complex, also led to a significant decrease in *10xSTAT92E-dGFP* activation (Fig. 2l,m). Thus STAT92E is genetically downstream of dAP-1 in the context of *10xSTAT92E-dGFP* reporter activation. Taken together, these genetic data argue that glia activate these transcriptional reporters after axonal injury through a TRAF4→JNK pathway→dAP-1→STAT92E signalling cascade that lies downstream of Draper.

### TRAF4 binds the Draper intracellular domain

The mechanism by which Draper activates JNK signalling and dAP-1-mediated transcriptional reporter activation has not been defined. The only molecule known to physically associate with Draper to promote downstream signalling events is the Src family kinase Shark, which binds an ITAM in the Draper intracellular domain after phosphorylation by Src42a (ref. 4). We found that depletion of Shark from all glia by pan-glial expression of *UAS-shark*^*RNAi*^ (using the *repo-Gal4* driver) blocked activation of *TRE-eGFP* 1 day after axotomy (Supplementary Fig. 5a,b), and similar RNAi-mediated knockdown of *rac1* or *src42a* also significantly decreased injury-induced activation of *TRE-eGFP* (Supplementary Fig. 5c,d). We therefore conclude that dAP-1 transcriptional reporters are genetically downstream of Draper/Shark signalling interactions.

**Figure 3:**
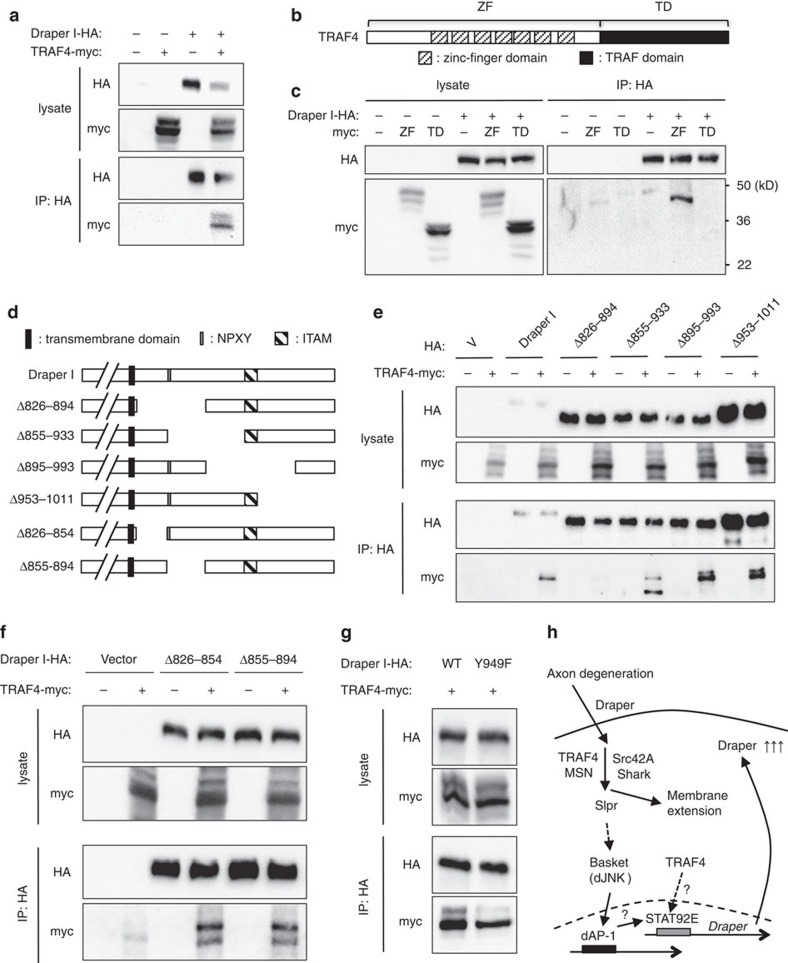
TRAF4 physically associates with Draper independently of Shark. (**a**) Western blots of co-immunoprecipitations from *Drosophila* S2 cells showed TRAF4-myc associated with Draper I-HA. All the blots shown are representative images after three repeats. (**b**) Schematic drawing of *Drosophila* TRAF4. ZF: zinc-finger enriched domain. TD: TRAF domain. (**c**) Draper I-HA interacted with the ZF region of TRAF4 but not the TRAF domain. (**d**) Schematic drawing of deletions in the Draper I intracellular for different constructs. The extracellular domain (//) was not drawn to scale. All the Draper I constructs were C-terminally tagged with HA. (**e**) The deletion of a.a. 826-894 decreased the association between Draper I and TRAF4. V: vector alone. (**f**) Draper I Δ826-854 and Δ855-894, bound TRAF4-myc at similar levels, suggesting these two regions are equally important in mediating the interaction between TRAF4 and Draper I. (**g**) The mutation Y949F in Draper, which is required for Shark to bind Draper I, did not affect the interaction between Draper I and TRAF4, indicating that the interaction between TRAF4 and Draper I does not require Shark association. (**h**) Proposed model of Draper-mediated glial transcriptional responses to axon degeneration. Draper is cell-autonomously required for glia to respond to axon degeneration and genetically upstream of JNK signalling cascade activation. The two binding partners of Draper, TRAF4 and Shark, are also upstream of JNK signalling cascade, likely through the activation of Slpr. We propose activation of JNK signalling cascade induces dAP-1-mediated gene transcription, which is also upstream of STAT92E-dependent transcriptional activation. The activation of STAT92E leads to the upregulation of Draper and facilitation of glial engulfment of axonal debris.

Given that TRAF4 is an adaptor protein, we speculated that TRAF4 might interact with Draper and thereby directly couple Draper to activation of JNK kinase signalling. TRAF4/MSN have well-defined roles in the activation of Slipper (ref. 20), and in turn the *Drosophila* JNK signalling pathway. We therefore performed co-immunoprecipitation (co-IP) experiments using *Drosophila* S2 cell lysates with a Myc-tagged TRAF4 (TRAF4-myc) and HA-tagged Draper I (Draper-I-HA). We found that pull down of Draper-I-HA led to the co-precipitation of TRAF4-myc, indicating that TRAF4 can physically associate with the Draper signalling complex (Fig. 3a). To identify the region through which TRAF4 associates with Draper we assayed the ability of epitope-tagged versions of the N-terminal TRAF4, which is enriched with zinc-finger domains (ZF, aa 1-927) or the C-terminal TRAF domain (TD, aa 928-1458)^19^ to associate with Draper. We found that the N-terminal TRAF4 domain co-immunoprecipitated with Draper I-HA, while the C-terminal TRAF domain, which is known to bind MSN (ref. 19), was not detected in the same assay (Fig. 3b,c). We therefore conclude that Draper is bound by the N-terminal domain of TRAF.

To identify the region of Draper-I to which TRAF4 bound, we next made serial deletions in the intracellular domain (Fig. 3d), and performed co-IP with full-length TRAF4-myc. We found that aa 826-894 were essential for Draper-I-TRAF4 interactions (Fig. 3e, Δ826-894), while all other regions were dispensable. We note this includes the evolutionarily conserved NPXY motif that is likely bound by the PTB domain binding protein dCED-6 (ref. 23), which is consistent with the observation that dCED-6 is not required for activation of *10xSTAT92E-dGFP* (ref. 6) or JNK signalling (Supplementary Fig. 6) in glia after axotomy. Further analysis of this subdomain revealed that aa 826-854 and 855-594 were both able to bind TRAF4 (Fig. 3d,f), suggesting there may be multiple TRAF4 binding sites in the Draper intracellular domain. The NPXY motif is not absolutely required for TRAF4 binding, as TRAF4 associated strongly with the Δ855-894 construct lacking this domain (Fig. 3f). Finally, given that both Src42A and Shark are required for activation of *TRE-eGFP* in response to injury, we assayed whether TRAF4 and Shark binding were reciprocally interdependent. Phosphorylation of Y949 in ITAM, which is mediated by Src42A and is bound by Shark (ref. 4), was also dispensable for TRAF4 to associate with Draper (Fig. 3g). Reciprocally, elimination of the TRAF4 interaction domain in Draper I (Δ826-894) did not alter Shark association with Draper (Supplementary Fig. 7). Taken together these data indicate that TRAF4 physically associates with the Draper intracellular domain in the region of the NPXY motif, and this interaction occurs in a Shark-independent fashion. These data argue Draper activates the JNK signalling pathway and dAP-1- and STAT92E-dependent transcriptional reporters in response to axon degeneration by direct association with TRAF4 and activation through MSN.

## Discussion

Activation of JNK/AP-1 and STAT transcriptional activity during reactive glial responses is an evolutionarily conserved feature of *Drosophila* and mammalian glia^24^,^25^. However, transmembrane receptors on glia that receive extracellular injury signals and in turn activate AP-1 or STAT-dependent transcriptional changes have not been identified. Our work supports a model whereby the engulfment receptor Draper is activated in response to axonal injury, which in turn promotes JNK signalling through TRAF4 and MSN to alter glial transcription via dAP-1 and STAT92E. Our reliance on well-characterized transcriptional reporters for dAP-1 and STAT92E supports this conclusion, but we note that we have not formally shown transcriptional activation of a downstream target gene, primarily because none have been identified.

Axon degeneration is required to activate glial cells after injury. Blockade of axon degeneration using *hiw* mutations fully suppresses glial responses to axon injury, including morphological changes and activation of transcriptional reporters, indicating that axonal self-destruction leads to the production of cues that activate glial responses through Draper. Interestingly, based on the activation of glial transcriptional reporters (that is *TRE-RFP*) even at a distance from the injury site (Fig. 1f), it appears that glia can be activated by degenerating axons even when not in direct contact with severed axons. This observation suggests that axonal ‘injury signals' that stimulate Draper signalling may be soluble, or, alternatively that glial-glial signalling may propagate through the glial network in a Draper-dependent manner. Our identification of Draper/TRAF4 interactions as a mechanism for JNK activation raises the intriguing possibility that MEGF10 might play a conserved role in mammalian reactive astrocytes to activate AP-1 or STAT signalling, or other contexts. Indeed, MEGF10 and several other engulfment genes are expressed in astrocytes and MEGF10 is required for developmental synapse pruning^26^.

We propose a simple model where axon self-destruction leads to injury signals that activate Draper, which in turn drives two primary downstream signalling events (Fig. 3h). In the first, Draper promotes cytoskeletal rearrangements essential for phagocytic activity through Src42a, Shark and Traf4 in direct association with Draper, and downstream GEF complexes such as Crk-II/Mbc/dCed-12 and DRK/DOS/SOS (refs 5, 27). These signalling pathways mediate membrane extension to, and internalization of engulfment targets. At the same time we propose that Draper activation leads to glial transcriptional responses to injury, including upregulation of dAP-1 and STAT92E transcriptional targets (ref. 12), although these need to be identified and validated. Consistent with the *draper* gene being a primary transcriptional target of this cascade, re-expression of Draper in glia is sufficient to rescue engulfment defects after knockdown of JNK, dAP-1 and STAT92E (refs 6, 7). However, re-expression of Draper is not sufficient to rescue engulfment defects in animals lacking glial Src42a or TRAF4 (Supplementary Fig. 4). The simplest interpretation of these data is that Draper, TRAF4 and Shark function upstream in the signalling pathway and are required for both internalization of targets and activation of transcriptional responses. This model is supported by our finding that TRAF4 and Shark directly bind Draper. Depletion of TRAF4 or STAT92E from glia alters basal levels of Draper, while, surprisingly, RNAi mediates suppression of dJNK or dAP-1 does not. This may indicate additional signalling pathways promote cross-talk between TRAF4 and STAT92e during transcriptional regulation of basal levels of *draper*, which is independent of dJNK.

Draper/MEGF10-mediated signalling through TRAF4 may also be engaged during developmental synapse elimination or reactive gliosis in mammals, but this awaits further exploration. Draper/MEGF10 has recently been shown to have potentially non-phagocytic signalling roles including activation of autophagy in *Drosophila* salivary glands^28^ and the tiling of starburst amacrine cells ref. 17. Exploring the roles for TRAF4, dAP-1 and STAT could provide further important molecular insight into Draper/MEGF10 regulation of transcription changes in these physiological contexts.

## Methods

### Fly strains

*Drosophila melanogaster* strains used in this study include: *OR85e-mCD8::GFP/CyO* (ref. 29), *repo-Gal4/TM3* (ref. 30), *nSyb-Gal4, 5xUAS-mCD8::GFP* (ref. 31), *UAS-traf4^IR^* (gift from T. Xu, Yale U)^18^, *UAS-drprI(24127)-HA, TRE-eGFP-16* (ref. 13), *TRE-RFP(dsRed.T4)-16* (ref. 13), *tub-Gal80^ts^, OR85e-mCD8::GFP/CyO* (ref. 27), *UAS-msn^DN^* (gift from M. Leptin, Cologne U, Cologne)^32^, *10xSTAT92E-GFP; repo-Gal4/TM3* (ref. 6), *10xSTAT92E-dGFP; repo-Gal4/TM3* (ref. 6), *UAS-puckered* (Bloomington Stock Center), *drpr^Δ5rec9^* (ref. 33), *repo-FLP^6–2^* (II)^34^, *FRT2A, FRT82B* (III)^33^, re*po-Gal4, UAS-mCD8::GFP/TM3* (ref. 6), *hiw^ΔN^* (ref. 35), *UAS-gfp::hiw* (ref. 31), *tub-Gal80^ts^; repo-Gal4, UAS-mCD8::GFP/TM3* (ref. 27), *UAS-Rac1^N17^*(ref. 36) and *UAS-shark^RNAi#6b^* (ref. 4). The following *Drosophila* strains were generated during the study according to standard procedure: *TRE-eGFP-16, tub-Gal80^ts^* (II), *drpr^Δ5rec9^, FRT2A/TM6, TRE-RFP-16, UAS-mCD8::GFP (II), tub-Gal80, FRT2A, repo-Gal4/TM3, UAS-drprI-HA, drpr^Δ5rec9^/TM6, repo-Gal4, drpr^Δ5rec9^/TM6 *and *hiw^ΔN^; UAS-hiw* & *UAS-gfp-hiw* (ref. 35). *UAS-src42A^RNAi#26019^*, *UAS-basket^RNAi#34138^* and *UAS-kayak^RNAi#6212^* were obtained from Vienna Drosophila Resource Center.

### Antibodies

For primary antibodies used in this study: Mouse anti-GFP clone 3E6 (Molecular Probes A-11120 1:200 for IFA; 1:2000 for WB), rabbit anti-Draper (ref. 37) (1:500 for IFA and 1:1000 for WB after pre-absorbed with *yw* embryos overnight), mouse anti-α-tubulin (DM1A) (Sigma T9026, 1:1000), rat anti-mCherry (Invitrogen M11217, 1:1000), rat anti-HA (3F10) (Roche 1-867-423, 1:2000 for WB), mouse anti-myc (9E10) (Millipore 05-419, 1:5000), mouse anti-Hiw (6H4) (Developmental Studies Hybridoma Bank, 1:1000) and mouse anti-Repo (Developmental Studies Hybridoma Bank, 1:5). FITC anti-mouse IgG (715-095-150), Cy3 anti-rabbit IgG (711-165-152), Cy3-anti-rat IgG (712-1165-150), Cy3 anti-mouse IgG (715-165-150) and Cy5 anti-rabbit IgG antibodies (711-165-152) were from Jackson ImmunoResearch Laboratories (all 1:100), as well as horseradish peroxidase-conjugated anti-rat antibodies (712-035-150, 1:6000). Both horseradish peroxidase-conjugated anti-mouse (ab6808), and anti-rabbit IgG (ab6721-1) were from Abcam (1:6000).

### Injury protocol and adult brain dissection

*Drosophila* cultures were kept at 25 °C except for the experiments involving *UAS-msn*^*DN*^ and *UAS-src42A*^*RNAi#26019*^, due to the lethality they caused when overexpressed using *repo-Gal4*. Temperature-sensitive GAL80 (*Gal80*^*ts*^)^38^ was used to inhibit the activity of GAL4 at 18 °C before fly eclosion and then deactivated at 29 °C for at least 5 days before injury. Flies continued to grow at 29 °C until the day of dissection. Maxillary palp and the third antennal segment ablation were performed using a pair of fine forceps under dissection microscope. For immunocytochemistry, heads were first fixed and permeabilized in 4% formaldehyde/0.1% Triton X-100/PBS for 20 min at room temperature (R.T.). After washed with PBST (0.1% Triton X-100/PBS) for five times, brains were dissected in PBST and post-fixed with 4% formaldehyde/0.1% Triton X-100/PBS for another 15 min. Fixed brains were then washed for five times with PBST, blocked in 0.1% Triton X-100/5% BSA/PBS for 30 min at R.T., and incubated with primary antibodies in PBST at 4 °C overnight or two nights for anti-Draper antibody. The next day, the brains were washed five times with PBST and then incubated with secondary antibodies in PBST for 2 h at R.T. or 4 h for Draper staining. After washed with PBST for five times, the brains were then mounted with VECTASHIELD Mounting Medium (Vector Labs) and stored at 4 °C before imaging within 2 weeks. For western blot analysis, heads from adult flies aged at 25 °C for at least 5 days after eclosion were isolated and immediately dissected in chilled PBS. Only the central brains (without the optic lobes) were collected and prepared for western blot analysis. Equal number of males and females were used for each experiment. Dissected brains were spinned down at the highest speed for 1 min and then lysed in 1X Laemmli lysis buffer (2 μl per brain) on ice for 20 min. The mixture was ground for 30 s on ice and centrifuged at the highest speed for 10 min at 4 °C. Only the supernatants were collected for SDS-PAGE analysis and western blotting. Images have been cropped for presentation in Fig. 2i. Full-size images are presented in Supplementary Fig. 8.

### Confocal microcopy and image analysis

Confocal microscopy settings were always kept constant throughout the same set of experiments. Zeiss LSM5 Pascal confocal microscope was used for all axonal debris clearance, TRE-eGFP reporter (except for *UAS-msn*^*DN*^), and STAT92E reporter assays with Zeiss Plan-Apochromat × 63/1.4NA oil objective lens. Glial MARCM clones, glial activation in *hiw*^*ΔN*^ animals and TRE-eGFP reporter activity in *msn*^*DN*^ animals were analysed using Zeiss Axio Imager.M2 spinning-disk confocal microscope with Zeiss C-Apochromat × 40/1.2NA water objective lens. For quantification of the fluorescence intensity, the centre single z-section was identified from each image stack of the relevance glomerulus or central adult brain, and pixel intensity was measured using ImageJ (National Institute of Health).

### DNA cloning and Draper I intracellular domain deletions

To clone TRAF4 cDNA, forward primer containing KpnI and reverse primer containing SacII restriction site (all the primer sequences are listed in Supplementary Table 1) were used to amplify TRAF4 cDNA from BDGP (Berkeley Drosophila Genome Project) cDNA clone LD20987. An approximate 1.6 kb cDNA was amplified by Phusion High-fidelity DNA polymerase (New England BioLabs), purified with QIAquick PCR Purification (QIAGEN) and then digested by KpnI and SacII. Digested DNA fragment was ligated to pUAST-CT-myc vector that has 6x myc sequence (MEQKLISEEDLNE) at the C-terminus, using T4 DNA ligase (New England BioLabs). The N-terminal (ZF, a.a. 1-309) and the C-terminal TRAF domain (TD, a.a. 310-494) of TRAF4 were isolated from pUAST-TRAF4-myc using the primers listed in Supplementary Table 1. Amplified PCR products were digested with both BglII and SacII, respectively, and then ligated to pUAST-CT-myc vector. Internal deletions in the Draper I intracellular domain were generated using GeneArt Seamless Cloning and Assembly Enzyme Mix (Life Technologies). See Supplementary Table 1 for the exact sequences of the primers used to amplify Draper I fragments from pAc5-DraperI-HA. pAc5 vector was linearized by KpnI and XbaI, purified with QIAquick PCR Purification (QIAGEN), and then incubated with purified Draper I-HA fragments in 1x seamless enzyme mix at R.T. for 15 min followed by standard bacterial transformation procedure. To make C-terminally truncated Draper I (Δ953-1101), the forward primer (containing a KpnI restriction site and the sequences matching Draper I nucleotide (nt.) 1-16), and the reverse primer (containing a XbaI restriction site, a stop codon (TAG), 1x HA DNA sequence and Draper I nt. 2838-2856) were used to amplify Draper I cDNA from pAc5-Draper I. PCR product was purified and digested with KpnI and XbaI, and then ligated to pAc5 vector. All the restrict enzymes used were purchased from New England BioLabs.

### Schneider 2 cell culture and co-immunoprecipitation

*Drosophila* S2 cells were cultured and maintained in 10% HyClone Fetal Bovine Serine/SFX-Insect Media (Fisher Scientific) with 1% penicillin-streptomycin solution (Sigma-Aldrich) at 25 °C. For immunoprecipitation of Draper I-HA, 2 × 10^6^ S2 cells were seeded per well in a six-well plate the day before DNA transfection, where 100 ng of each appropriate DNA vector were mixed and incubated with Effectene Transfection Reagents (QIAGEN), as described in manufacturer's instruction. Two days after transfection, cells were lysed in our modified NP-40 lysis buffer (1% NP-40, 10 mM Tris-HCl pH 7.5, 50 mM NaCl, 30 mM Na_4_P_2_O_7_, 50 mM NaF, 100 μM Na_3_VO_4_, 5 μM ZnCl_2_ and protease inhibitor cocktail purchased from Roche)^12^. Rat anti-HA monoclonal antibody (0.3 μg per sample) was incubated with Protein G beads (Sigma-Aldrich) at 4 °C for 3 h before incubated with pre-cleared cell lysates at 4 °C overnight. After washed at least eight times to reduce non-specific binding, proteins on beads were eluted in SDS loading buffer (60 mM Tris pH 6.8, 10% glycerol, 2% SDS, 1% β-mercaptoethanol and 0.01% bromophenol blue) and boiled at 100 °C for 5 min, following a 2-min centrifugation at 16,000 g. Supernatants were loaded into 4–15% gradient Tris-HCl ReadyGel (Bio-Rad). SeeBlue Pre-stained Protein Standard (Life Technologies) was used (10 μl per well) to determine protein molecular weight. Samples were transferred to nitrocellulose membrane (Bio-Rad) after electrophoresis. Before incubated with corresponding primary antibodies at 4 °C overnight, membrane was blocked in 5% milk/0.01% Tween-20/PBS at R.T. for 1 h. Membrane was then washed with 0.01% Tween-20/PBS two times in 30 min and then probed with corresponding secondary antibodies at R.T. for 1 h, followed by another two washes in 30 min. Immunoreactivity was detected using Clarity Western ECL Substrate (Bio-Rad) or ECL Prime Western Blotting Detection Reagent (Amersham) for TRAF4-myc after co-IP. Image acquisition and protein quantification were conducted using ChemiDoc^TM^ MP system (Bio-Rad) and signal saturation was avoided during acquisition. Images have been cropped for presentation in Fig. 3a,c,e–g. Full-size images are presented in Supplementary Fig. 8. All results showed were representative blots after at least three repeats.

### Statistical analysis

All error bars represent s.e.m. Unpaired Student's *t*-test (2-tailed), one-way ANOVA, and post-hoc analyses were carried out using GraphPad Prism 6 (GraphPad Software). Data distribution was assumed to be normal, although this was not formally tested. No statistical methods were used to predetermine sample sizes, but our sample sizes are similar to those reported in previous publications. Also, data collection and analysis were not performed blind owing to the conditions of the experiments. Data were not collected and processed randomly. Animals were assigned to the various experimental groups on the basis of genotype.

### Data availability

The authors declare that the experimental results supporting the findings are included in the article and the Supplementary Information or are available upon request.

## Additional information

**How to cite this article:** Lu, T.-Y. *et al*. Axon degeneration induces glial responses through Draper-TRAF4-JNK signalling. *Nat. Commun.* **8,** 14355 doi: 10.1038/ncomms14355 (2017).

**Publisher's note:** Springer Nature remains neutral with regard to jurisdictional claims in published maps and institutional affiliations.

## Supplementary Material

Supplementary Information Supplementary Figures and Supplementary Table.
