# Supplementary material for: Axon degeneration induces glial responses through Draper-TRAF4-JNK signalling
Source: Nat Commun. 2017 Feb 6;8:14355. doi: 10.1038/ncomms14355 (PMC5303877; doi:10.1038/ncomms14355)
Supplement: Supplementary Information — Supplementary Figures and Supplementary Table. [file ncomms14355-s1.pdf]

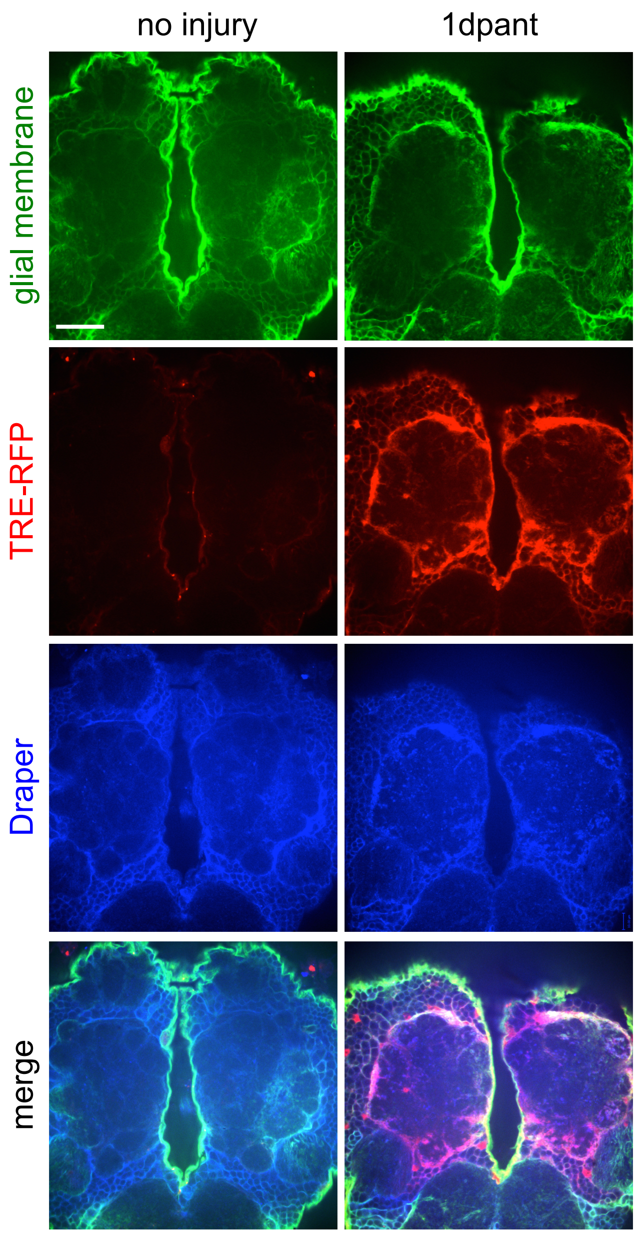

Supplementary Figure 1. Axon injury robustly activates the *TRE-RFP* reporter in glia throughout the brain. Glial membranes surrounding the antennal lobe region were genetically labeled by membrane GFP (mCD8::GFP), the expression of which was driven by pan-glial driver *repo-Gal4*. The *TRE-RFP* reporter activity was undetectable in the uninjured brains (no injury) by anti-mCherry antibodies. One day after the ablation of the third antennal segments (1dpant), a massive amount of axons in the antennal lobe underwent Wallerian degeneration, as indicated by glial membrane hypertrophy and upregulation of Draper (anti-Draper, blue) near the antennal lobe. Meanwhile, a robust increase of *TRE-RFP* reporter activity was observed not only surrounding the antennal lobe, but also in glia that were remote from the antennal lobe at the edge of the brain. Representative, single-plane confocal images were shown from at least 10 animals. Animal genotype: *hiw*$^{\Delta N}$*/+; TRE-RFP-16/+; repo-Gal4, UAS-mCD8::GFP/+*. Scale bar = 50 µm.

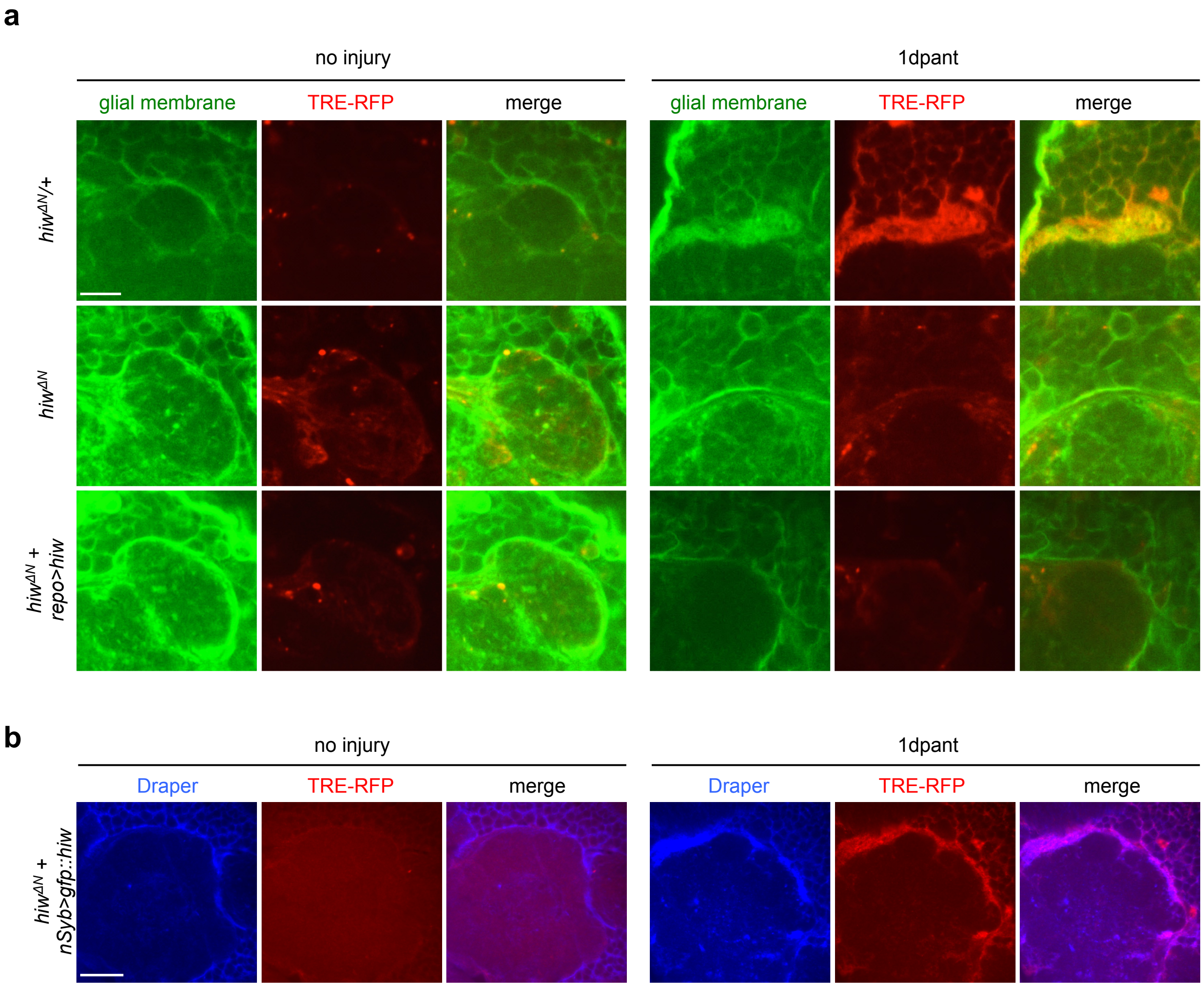

Supplementary Figure 2. Glial responses to axon degeneration requires Hiw in neurons, but not in glia. a. In *hiw*$^{\Delta N}$ mutants, re-expression of Hiw in glial cells did not rescue glial activation of membrane hypertrophy and the activation of the *TRE-RFP* reporter 1 day after antennal ablation in *highwire* null animals. Representative images were shown from 3 animals each. *hiw*$^{\Delta N}$*/+*: *hiw*$^{\Delta N}$*/+; TRE-RFP-16/+; repo-Gal4, UAS-mCD8::GFP/+*. *hiw*$^{\Delta N}$: *hiw*$^{\Delta N}$*/Y; TRE-RFP-16/+; repo-Gal4, UAS-mCD8::GFP/+*. *hiw*$^{\Delta N}$ + *repo>hiw*: *hiw*$^{\Delta N}$*/Y; TRE-RFP-16/UAS-hiw; repo-Gal4, UAS-mCD8::GFP/+*. Scale bar = 10 μm. **b.** In *hiw*$^{\Delta N}$ mutants, re-expression of Hiw in neurons restored glial upregulation of Draper expression and the activation of the *TRE-RFP* reporter. Representative images were shown from 6 animals each. *hiw*$^{\Delta N}$ + *nSyb>hiw: hiw*$^{\Delta N}$*/Y; TRE-RFP-16/+; nSyb-Gal4, 5xUAS-mCD8::GFP/5xUAS-gfp-hiw*. Scale bar = 20 μm.

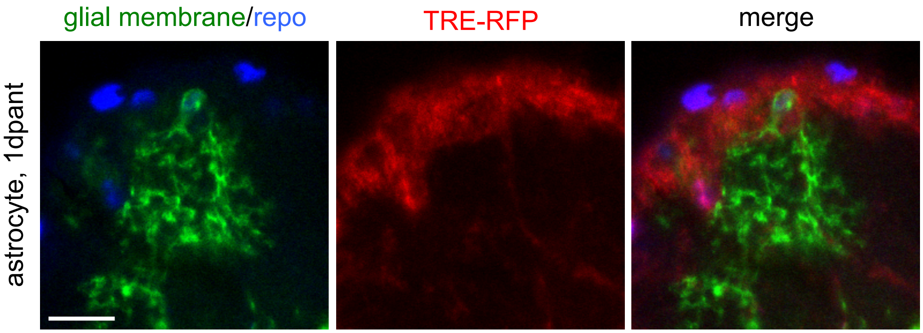

Supplementary Figure 3. Astrocytes do not activate JNK signaling cascade after axon injury. A wild-type astrocyte MARCM clone was labeled with mCD8::GFP (green). Anti-repo antibody (blue) labeled glial nuclei. At day 1 post antennal ablation, no TRE reporter activation (red) was observed in astrocytes. Representative images were shown from at least 10 animals. Animal genotype: *TRE-RFP-16, UAS-mCD8::GFP/repo-FLP*$^{6\text{-}2}$*; FRT2A, FRT82B/tub-Gal80, FRT2A, repo-Gal4*. Scale bar = 10 μm.

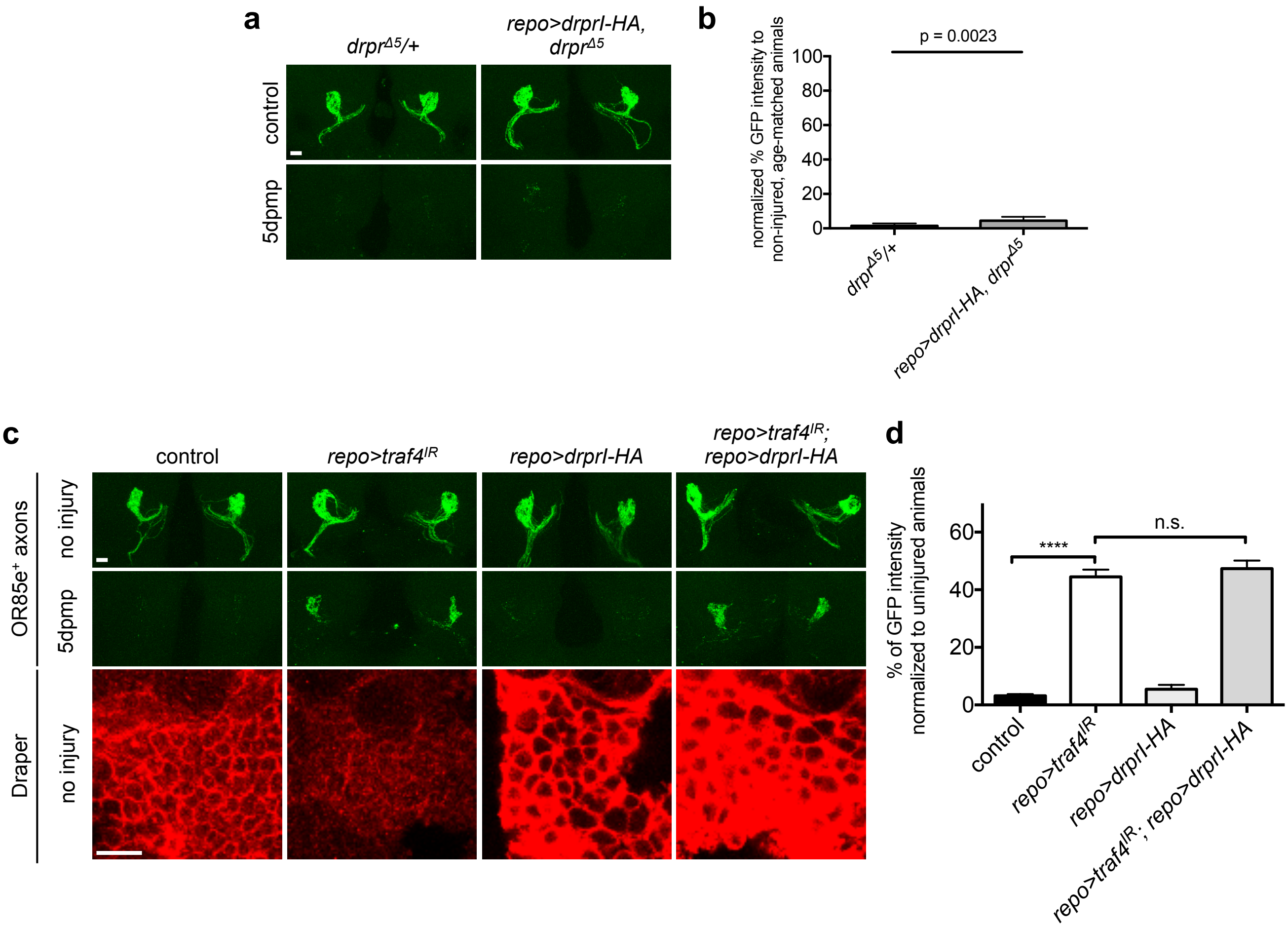

Supplementary Figure 4. TRAF4 is necessary for glia to engulf axonal debris. a. Draper I-HA functionally rescued the clearance defect in the *draper* null (*drpr*$^{\Delta5}$) animals. In *drpr*$^{\Delta5}$ animals, more than 80% of the OR85e^{+} axonal debris was uncleared 5 days after maxillary palp ablation (5dpmp)[2,27]. However, when *UAS-Draper I-HA* expression was driven by *repo-Gal4*, the majority amount of axonal debris was cleared within 5 days after injury, comparable to *draper* heterozygous null animals (*drpr*$^{\Delta5}$/+) where no clearance defect was observed. Representative images were shown from 10 animals each. *drpr*$^{\Delta5}$/+: *OR85e-mCD8::GFP/CyO; drpr*$^{\Delta5}$*/TM6B, Tb, Hu, e*. *Repo>drprI-HA, drpr*$^{\Delta5}$/+: *OR85e-mCD8::GFP/+; repo-Gal4, drpr*$^{\Delta5}$*/UAS-drprI-HA, drpr*$^{\Delta5}$. Scale bar = 10 μm. **b.** Quantification of data in a. Unpaired t-test, 2-tailed. *n* = 10 each. **c.** Draper I-HA did not rescue the clearance defect caused by *traf4*^{IR}. Representative images of OR85e^{+} axonal materials in the antennal lobe and Draper immunoreactivity in the cortex region of the same brain were shown from at least 10 animals. Control: *OR85e-mCD8::GFP/+; repo-Gal4/+*. *Repo>traf4*^{IR}: *OR85e-mCD8::GFP/UAS-traf4*^{IR}*; repo-Gal4/+*. *Repo>drprI-HA*: *OR85e-mCD8::GFP/+; repo-Gal4/UAS-drprI-HA*. *Repo>traf4*^{IR}; *repo>drprI-HA*: *OR85e-mCD8::GFP/UAS-traf4*^{IR}*; repo-Gal4//UAS-drprI-HA*. Scale bar = 10 μm. **d.** Quantification of OR85e^{+} axonal materials remained in c. 5 days after injury. *n* = 20 for control and 10 for others each. n.s.: not significant. One-way ANOVA. Tukey's post-hoc.

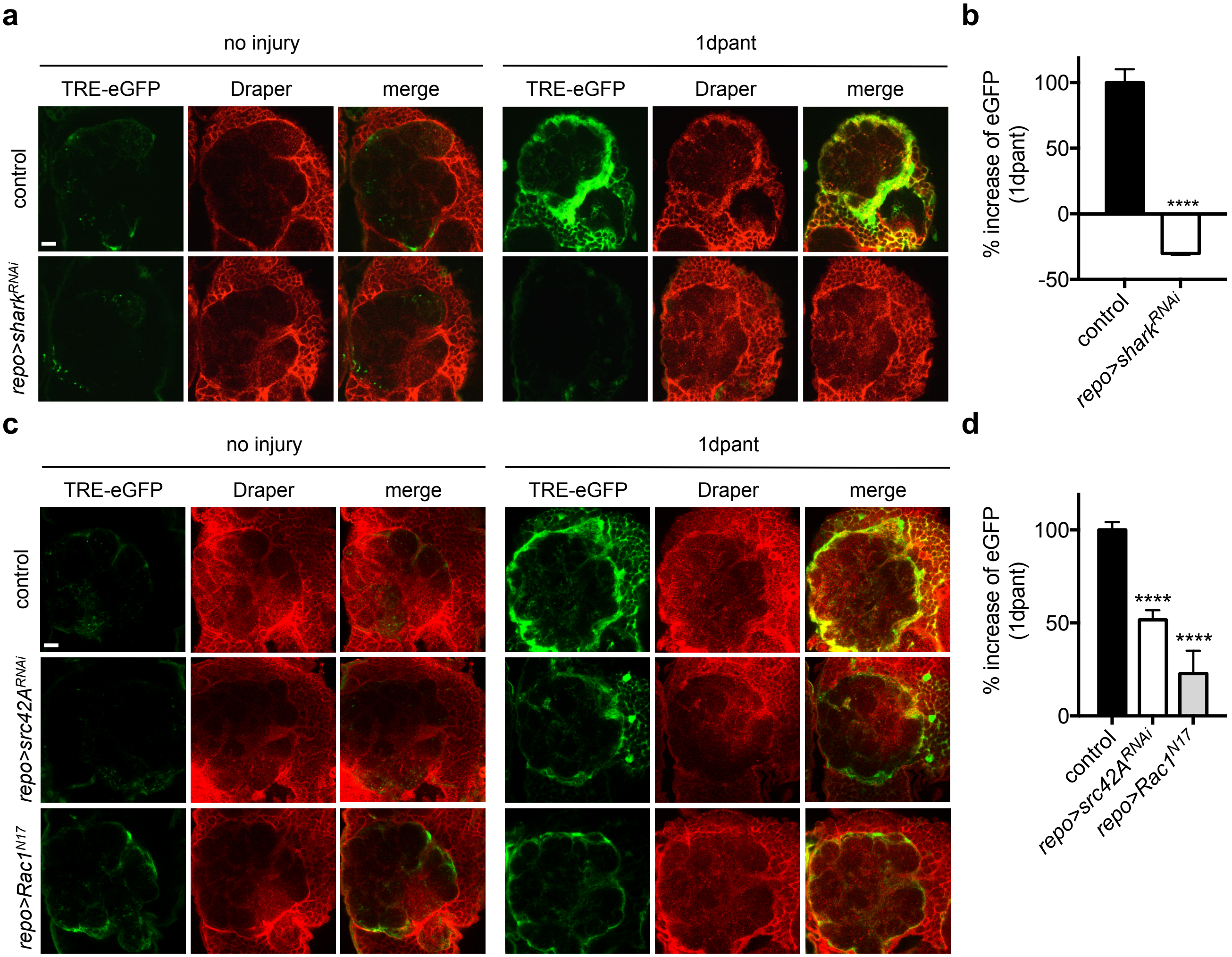

Supplementary Figure 5. Src42A, Rac1 and Shark are required for glia to activate JNK signaling cascade in response to axon degeneration. a. Knocking down Shark in glia (*shark*^{RNAi}) almost completely suppressed the activation of the *TRE-eGFP* reporter 1 day after antennal ablation. Control: *TRE-eGFP-16/+; repo-Gal4/+*. *Repo>shark*^{RNAi}: *TRE-eGFP-16/+; repo-Gal4/UAS-shark*$^{RNAi\#6b}$. Representative images were shown from at least 3 different animals. Scale bar = 10 µm. **b.** Quantification of the increase of eGFP immunoreactivity in c. $n = 15$ each. Unpaired t-test, 2-tailed. **c.** Knocking down Src42A (*src42A*^{RNAi}) and blocking Rac1 activity (*Rac1*^{N17}) in glia suppressed the *TRE-eGFP* reporter activation 1 day after antennal ablation. Representative images were shown from at least 3 different animals. Control: *TRE-eGFP-16, tub-Gal80*^{ts}*/+; repo-Gal4/+*. *Repo>src42A*^{RNAi}: *TRE-eGFP-16, tub-Gal80*^{ts}*/UAS-src42A*$^{RNAi\#26019}$*; repo-Gal4/+*. *Repo>Rac1*^{N17}: *UAS-Rac1*^{N17}*; TRE-eGFP-16, tub-Gal80*^{ts}*/+; repo-Gal4/+*. Scale bar = 10 µm. **d.** Quantification of the increase of eGFP immunoreactivity in a. Control: $n = 8$. *Repo>src42A*^{RNAi}: $n = 7$. *Repo>Rac1*^{N17}: $n = 6$. One-way ANOVA and Bonferroni post hoc.

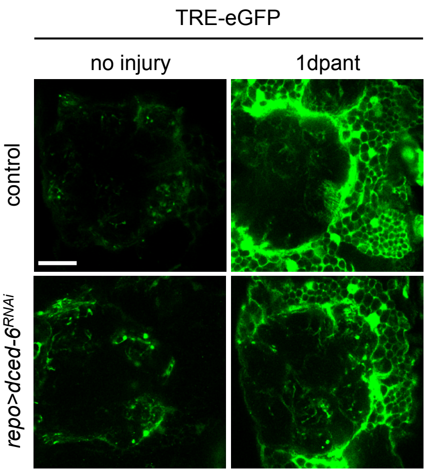

Supplementary Figure 6. dCed-6 is not required for glia to activate JNK signaling cascade after axonal injury. Knocking down dCed-6 using *UAS-dced-6^{RNAi}* construct does not cause discernable defect in the *TRE-eGFP* reporter activation 1 day after antennal ablation (1dpant). Representative images were shown from at least 3 different animals. Control: *TRE-eGFP-16, tub-Gal80^{ts}/+; repo-Gal4/+*. *Repo>dced-6^{RNAi}*: *TRE-eGFP-16, tub-Gal80^{ts}/dced-6^{RNAi}; repo-Gal4/+*. Scale bar = 20 µm.

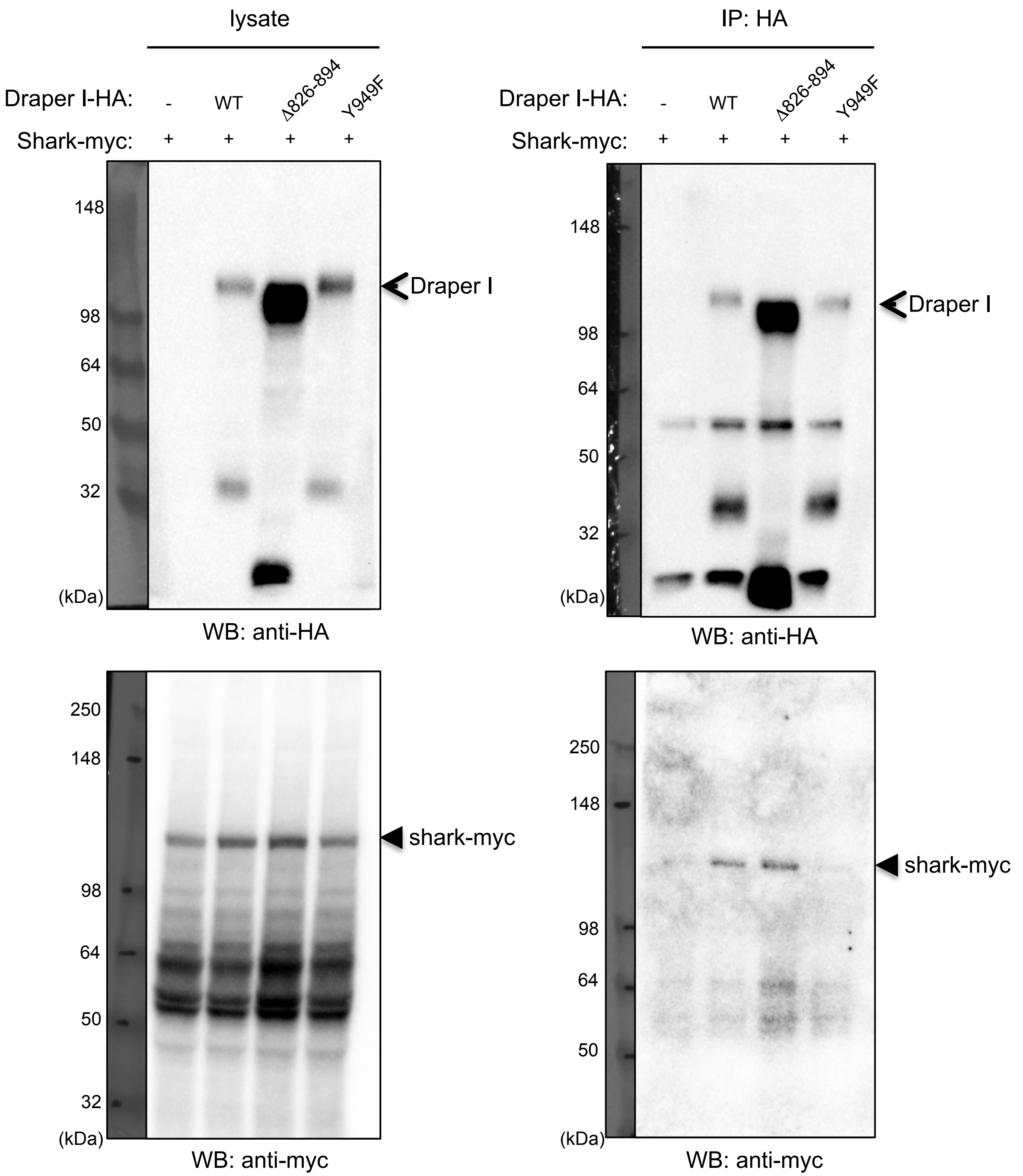

Supplementary Figure 7. The physical interaction between Draper I and TRAF4 is not required for Shark to bind Draper. Western blots from co-immunoprecipitation experiment using *Drosophila* S2 cell lysates transfected with different Draper I constructs and Shark-myc. WT: full-length Draper I. Δ826-894: The Draper I construct where amino acid no. 826 – 894 was deleted from the intracellular domain, and thus prevents TRAF4 from binding to Draper I (see Fig. 3). Y949F: The Draper I construct where the Tyr^{949} residue was mutated into phenylalanine to block Shark from binding to Draper I. Note that Shark-myc was co-immunoprecipitated with both WT and Δ826-894 construct but not Y949F.

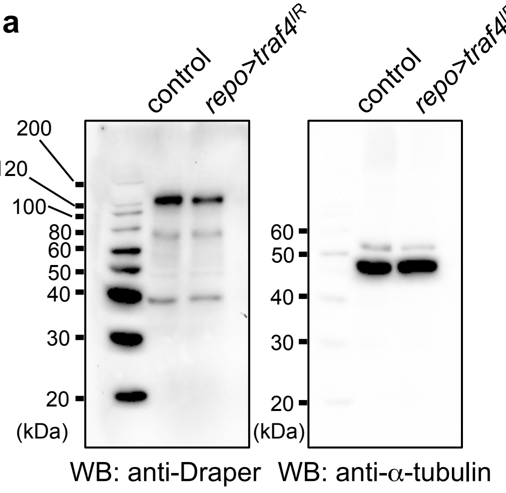

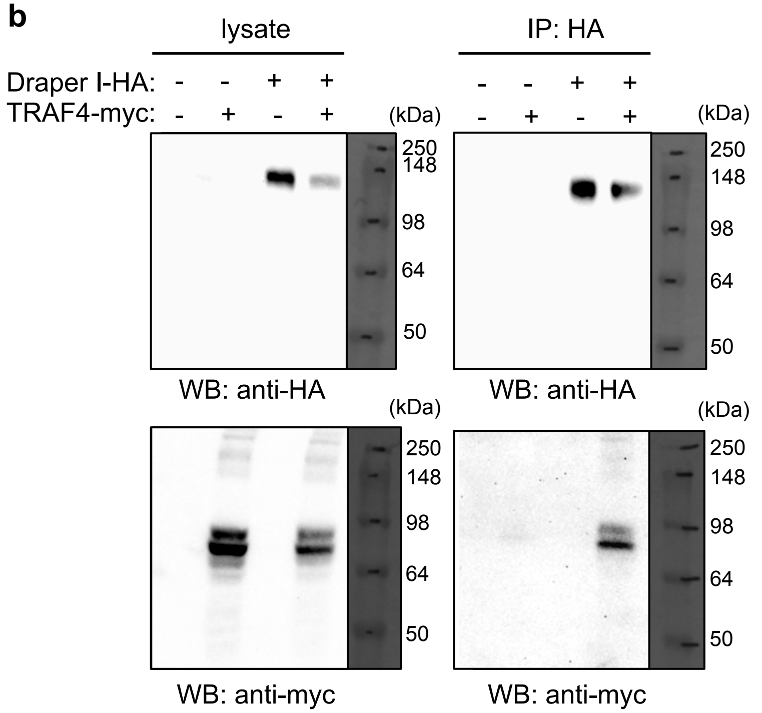

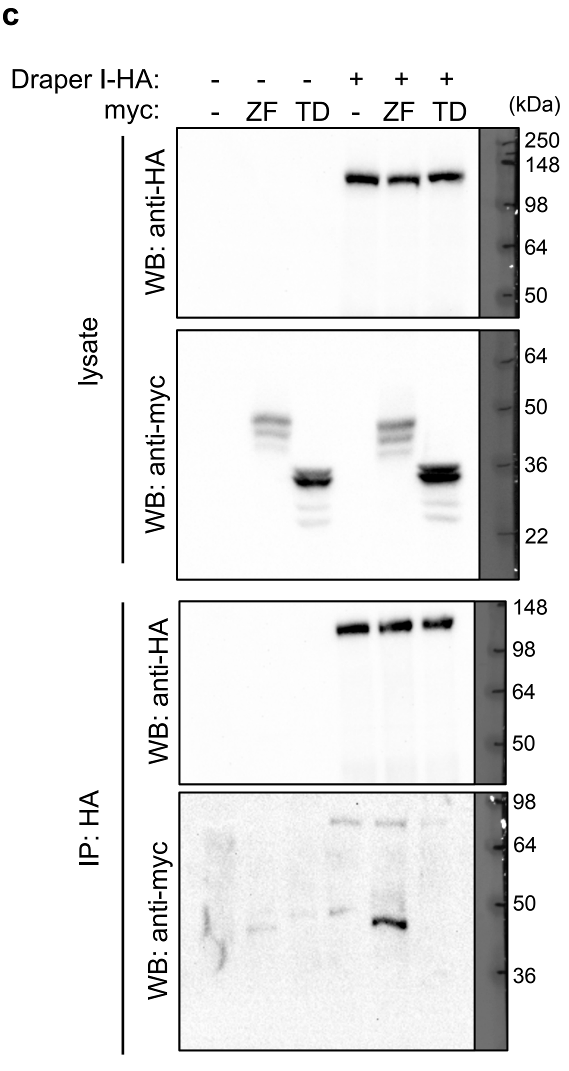

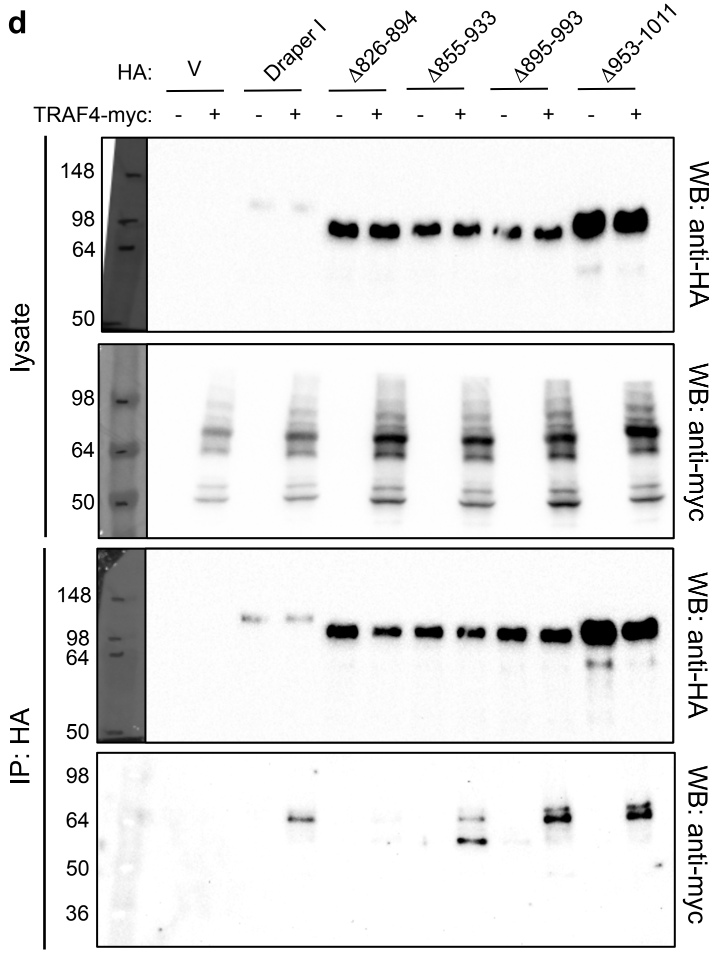

Supplementary Figure 8. Full-size Western blot images shown in the main figures, including Western blots in a. Figure 2i. **b.** Figure 3a. **c.** Figure 3c. **d.** Figure 3e. (Cont'd on next page.)

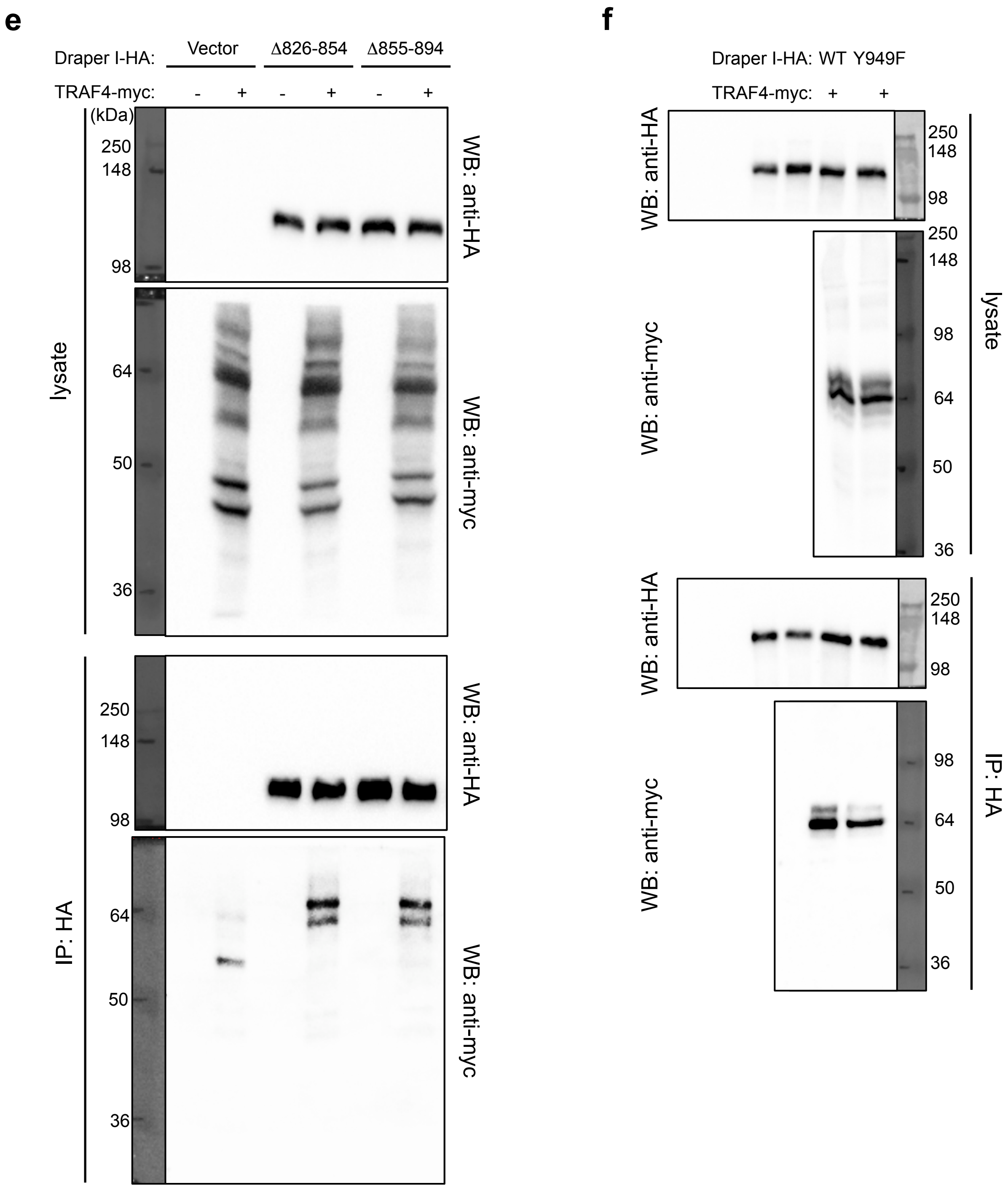

Supplementary Figure 8 (cont'd). **e.** Figure 3f. **f.** Figure 3g.

Supplementary Table 1. Primer sets used for cloning. Fw means forward primer. Rv means reverse primer. Restriction sites are underlined.

For cloning of	Primer sequence	Note
pUAST-TRAF4-myc	Fw: 5'- ttaaaggtaccATGGTTCGAAGTTTGGCCCAGTGGACG -3'<br>Rv: 5'- aattttaaaaccgcggGACGGCCACTATCTTGCTGG -3'	
pUAST-TRAF4(ZF)-myc	Fw: 5'- atcgagatctATGGTTCGAAGTTTGG -3'<br>Rv: 5'- aaaaccgcggCGAGCTGAGGGCCACCATCAGC -3'	a.a. 1-309
pUAST-TRAF4(TD)-myc	Fw: 5'- atcgagatctATGCGCCAGGGTCAGCAGATCC -3'<br>Rv: 5'- atcgccgcggGACGGCCACTATCTTGCTGG -3'	a.a. 310-494
pAc5-DraperI(Δ826-894)-HA	a.a. 1-825:<br>Fw: 5'- cccggatcggggtaccATGTTGCCGGTAATCC -3'<br>Rv: 5'- CTGCCACT ACGTCGGCGGTAGTA -3'<br><br>a.a. 895-1011:<br>Fw: 5'- GCCGACGT AGTGGCAGGGTGGGT -3'<br>Rv: 5'- gaagggccctctagaCTAAGCGTAATCTGG -3'	
pAc5-DraperI(Δ855-933)-HA	a.a. 1-854:<br>Fw: 5'- cccggatcggggtaccATGTTGCCGGTAATCC -3'<br>Rv: 5'- TCGTCGTA GTCGAAGTTGTGGTTG -3'<br><br>a.a. 934-1011:<br>Fw: 5'- ACTTCGAC TACGACGAGATCAAGCA -3'<br>Rv: 5'- gaagggccctctagaCTAAGCGTAATCTGG -3'	
pAc5-DraperI(Δ895-993)-HA	a.a. 1-894:<br>Fw: 5'- cccggatcggggtaccATGTTGCCGGTAATCC -3'<br>Rv: 5'- AGCGGTTT GGCATTGCAGTCATCG -3'	

	a.a. 994-1011:<br>Fw: 5’- GCAATGCC AAACCGCTGCCGCCC -3’<br>Rv: 5’- gaagggccctctagaCTAAGCGTAATCTGG -3’	
pAc5-DraperI(Δ953-1011)-HA	Fw: 5’- atcgggtaccATGTTGCCGGTAATCC -3’<br>Rv: 5’- aaaatctaga-CTAAGCGTAATCTGGAACATCATATGGGTAGGCCATCAGGTGATCGTATTCATCG -3’	
